# Modelling physiological deterioration in post-operative patient vital-sign data

**DOI:** 10.1007/s11517-013-1059-0

**Published:** 2013-03-21

**Authors:** Marco A. F. Pimentel, David A. Clifton, Lei Clifton, Peter J. Watkinson, Lionel Tarassenko

**Affiliations:** 1 Institute of Biomedical Engineering, Department of Engineering Science, University of Oxford, Old Road Campus Research Building (Off Roosevelt Drive), Oxford, OX3 7DQ UK; 2 Nuffield Department of Clinical Neurosciences, Oxford University Hospitals NHS Trust, Oxford, OX3 9DU UK

**Keywords:** Patient monitoring, Early warning scores, Novelty detection

## Abstract

Patients who undergo upper-gastrointestinal surgery have a high incidence of post-operative complications, often requiring admission to the intensive care unit several days after surgery. A dataset comprising observational vital-sign data from 171 post-operative patients taking part in a two-phase clinical trial at the Oxford Cancer Centre, was used to explore the trajectory of patients’ vital-sign changes during their stay in the post-operative ward using both univariate and multivariate analyses. A model of normality based vital-sign data from patients who had a “normal” recovery was constructed using a kernel density estimate, and tested with “abnormal” data from patients who deteriorated sufficiently to be re-admitted to the intensive care unit. The vital-sign distributions from “normal” patients were found to vary over time from admission to the post-operative ward to their discharge home, but no significant changes in their distributions were observed from halfway through their stay on the ward to the time of discharge. The model of normality identified patient deterioration when tested with unseen “abnormal” data, suggesting that such techniques may be used to provide early warning of adverse physiological events.

## Introduction

Delayed detection of clinical deterioration has been repeatedly associated with high rates of avoidable in-hospital death and intensive care unit (ICU) readmissions (which are associated with a substantially increased mortality rate) [8, 11, 15]. According to large national surgical audits such as the UK National Confidential Enquiry into post-operative deaths, current systems of post-operative care fail to detect or respond appropriately to early signs of critical illness [17]. Such failures have been explained by lack of experienced senior nursing staff, inexperienced trainee medical staff [17], poor quality of care offered to critically ill patients [6, 15], and, more importantly, the inability of current systems to recognise clinical deterioration early. All of these factors can lead to deterioration in a patient’s condition and admission to the ICU, or death.

The UK National Institute for Health and Clinical Excellence (NICE) [16] has recommended that physiological track and trigger (T&T) systems should be used to monitor all adult patients in acute hospital units, to promote the recognition of patient deterioration early enough to allow proper intervention by medical staff. These systems are based on early warning scores (EWS) calculated from the values of physiological variables observed periodically. Univariate scoring criteria are applied to each physiological variable (vital sign) in turn, and then care is escalated to a higher level if any of the scores assigned to individual vital signs, or the sum of all such scores, exceed some threshold. There is widespread interest and clinical utilisation of these scores in countries across Europe and Australasia, and increasingly in North America [5]. However, the quality of evidence supporting the use of T&T systems is poor [5], and they have a number of disadvantages. The thresholds and ranges of these EWS systems are mostly determined heuristically (although evidence-based methods have recently been proposed [18, 23]). Furthermore, each vital sign is treated independently and correlations between them are not taken into account. Also, the clinical setting from which data are acquired for either validating or designing the EWS system is an important consideration. Many studies have been conducted in medical assessment units [5, 18], and it is questionable whether the scores can be extrapolated to other medical units; for example, post-operative wards, general wards, or other settings.

An alternative approach to detecting patient deterioration from changes in vital signs is that of novelty detection [2, 20], or one-class classification, which involves the construction of a multivariate, multimodal model of normality using examples of “normal” vital signs. This then allows the classification of test data as either “normal” or “abnormal” with respect to that model. Several approaches to novelty detection have been proposed, and an extensive review of these techniques is presented in [13, 14]. We have shown how novelty detection can be combined with continuous vital-sign monitoring of acutely ill in-hospital patients [7, 9, 10, 21].

In this paper, we investigate models of normality tuned to a specific post-operative patient population, recovering from gastro-intestinal surgery. Following surgery, patients start in their most acute state and gradually stabilise. We hypothesise that models of the distribution of vital-sign data from “normal” patients may be used to describe the physiological trajectory associated with “normal” recovery of these patients. These models may then be used to identify “abnormal” trajectories in patients who experience major deterioration and have to be re-admitted to the ICU.

## Methods

### Dataset

Vital-sign data (heart rate, HR, measured in beats per minute; respiratory rate, RR, measured in breaths per minute; arterial blood oxygen saturation, SpO_2_, measured as a percentage; systolic blood pressure, SysBP, measured in mmHg; core temperature measured with a tympanic thermometer in °C; and a level of consciousness assessed typically with the Glasgow Coma Scale,^1^ GCS) were recorded by nursing staff during their regular observations of post-operative patients in the upper gastrointestinal (GI) ward at the Oxford Cancer Centre, Oxford University Hospitals NHS Trust, Oxford, UK. The dataset used for the work described by this paper comprises measurements of HR, RR, SpO_2_, SysBP and temperature (dimensionality of the input space, *D* = 5) acquired by ward staff every hour or every 2 h in the days immediately following the patient admission to the ward (depending on patient’s condition), and every 4 h in the last few days of the patient’s stay on the ward. These measurements were then transcribed by two independent research nurses into an electronic database.

**Fig. 1:**
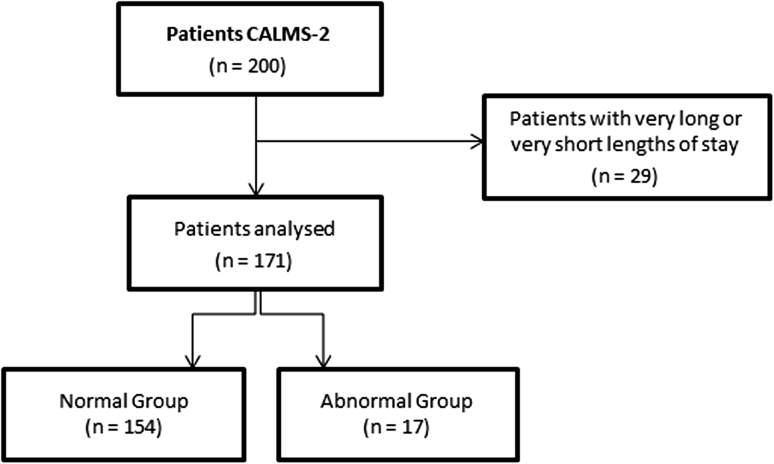
Flow diagram showing the steps involved in creating the “normal” and “abnormal” patient datasets, including the number of patients included in the study

200 patients were recruited during Phase I of the CALMS2 clinical trial in the upper GI ward (approved by the local research ethics committee, REC reference: 08/H0607/79). This dataset was firstly refined to include only observations with no missing physiological variables (for example, if an observation from a patient does not include HR, it was removed from the dataset). The median length of stay on the ward was 9 days, and we selected those patients who stayed on the ward for a minimum of 4 days (which corresponds to the 10th percentile) and a maximum of 29 days (90th percentile), to construct our model of normality, for the purposes of novelty detection. This reduces the number of patients from 200 to 171 (see Fig. 1).

From the original dataset, a set of 12,797 observations **X** ∈ ℝ^5^ obtained from the 171 patients was then analysed. From the patients analysed, those who were either admitted to the ICU or died on the ward were labelled as belonging to the “abnormal group” of patients (17 patients), while the remainder were labelled as being part of the “normal group” (154 patients). The patient population characteristics in each group are shown in Table 1. We note that the mortality rate in the “abnormal” set of patients was 35.3 %, which shows the severity of the risk associated with ICU re-admission.

**Table 1:** Patient demographics for the “normal” and “abnormal” groups of patients

	Normal group	Abnormal group
Number of patients	154	17
Number of observations	10,299	2,498
Age (mean ± SD)	61 ± 12	67 ± 10
Sex (male)	90 (58.4 %)	10 (58.8 %)
Length of stay (median ± IQR)	9 ± 5	5 ± 4^1^
Length of stay (25th/75th percentile)	7/12	4/8^1^
Mortality	0	6 (35.3 %)

^1^As described in Sect. 3.3, the “length of stay” for the abnormal group is the time to the first event (re-admission to ICU or death)

### Vital-sign distributions

The changes in vital-sign distributions between admission to the upper GI ward and subsequent discharge, when the patient was deemed sufficiently stable to go home, were evaluated for the patients in the “normal group”. Normalised histograms (unit area under the curve) and cumulative distribution functions (cdfs) were plotted for each physiological variable (HR, RR, SpO_2_, SysBP and temperature), using the average value for each variable on the admission and discharge days.

The trajectory of each vital sign throughout the patient’s stay on the ward was evaluated by examining the following subgroups of observations:

- G_1_: the set of averages of all observations performed on the first day of the patient’s stay on the ward (admission day);
- G_2_: the set of averages of all observations performed on the day that corresponds to a quarter (25 %) of the length of the patient’s stay on the ward;
- G_3_: the set of averages of all observations performed on the day that corresponds to half (50 %) of the length of the patient’s stay on the ward;
- G_4_: the set of averages of all observations performed on the day that corresponds to 75 % of the length of the patient’s stay on the ward;
- G_5_: the set of averages of all observations performed on the last day of the patient’s stay on the ward (discharge day).

These subgroups were defined in this way because of the different lengths of patient stay on the ward (which varied between 4 and 28 days). Three different metrics were used to compare the resulting vital-sign distributions: the Kolmogorov–Smirnov (KS) metric [4], the symmetrical Kullback–Leibler (KL) distance [12, 25] and the Bhattacharyya (*Bhat*) distance [1].

The KS distance is a non-parametric metric that quantifies the distance between the empirical distribution functions of two sample sets [4]. Considering two probability densities, *p* and *q*, if *P* and *Q* are the respective cdfs, the KS distance ($\Updelta KS$) between them is defined by

1 $$\Updelta KS(p,q) = \sup \left( {|P(x) - Q(x)|} \right) $$

where $\sup (y)$ is the supremum of the set of distances *y*.

The KL divergence [12] compares the entropy of two distributions over the same random variable. It measures the number of additional bits required when encoding a random variable with a distribution $p(x)$ using the alternative distribution $q(x)$. This measure is asymmetrical, but it can be modified to be the symmetrical KL distance ($\Updelta KL_{S}$) [25], defined as

2 $$\Updelta KL_{S} (p,q) = \sum\limits_{x \in X} {\left( {p(x) - q(x)} \right)\log \frac{p(x)}{q(x)}} $$

The *Bhat* distance ($\Updelta Bhat$) [1] measures the amount of overlap between two distributions, and is defined by

3 $$\Updelta Bhat(p,q) = - \log \left[ {BC(p,q)} \right] $$

where $$BC(p,q) = \sum\limits_{x \in X} {\sqrt {p(x)q(x)} }$$.

In order to study the physiological trajectory of the “normal” patients, the distributions of each vital sign, for each of the first 4 subgroups described (G_1_, G_2_, G_3_, G_4_) were compared with G_5_ (which contains the average of the vital signs from the most physiologically stable period of the patient stay), using the three metrics defined by (1), (2) and (3).

### Data visualisation

The first stage in constructing a model of normality for novelty detection usually consists of obtaining more insight into the structure of the data [22]. Procedures for visualisation of the data in their original high-dimensional space are therefore required.

Data in high-dimensional space ($D > 3$) can be visualised through a non-linear projection from ℝ^D^ to ℝ^2^. Sammon’s method [19] seeks to create a mapping such that the distances between pairs of image points in a projection plane ($y_{i} ,y_{j}$) are as close as possible to the distances between the corresponding pair of points in data space ($x_{i} ,x_{j}$). The following error function, known as the Sammon stress metric, is defined as

4 $$E = \frac{1}{{\sum\limits_{i < j} {d_{ij}^{*} } }}\sum\limits_{i < j}^{N} {\frac{{\left( {d_{ij}^{*} - d_{ij} } \right)^{2} }}{{d_{ij}^{*} }}} $$

with $d_{ij} = \left\| {x_{i} - x_{j} } \right\|$ and $d_{ij}^{*} = \left\| {y_{i} - y_{j} } \right\|$, where $\left\| \bullet \right\|$ is the Euclidean norm. The Sammon mapping aims to minimise the error metric (4), which can be achieved by initialising the image points *y* to have random locations in a 2-D map and by iteratively adjusting these locations in the direction which gives the maximum change in *E* using a gradient descent method.

It is assumed a priori that each of the five vital signs has equal importance in the model of normality. Each variable was therefore scaled to have approximately the same dynamic range to ensure that variables with large changes (e.g., blood pressure in mmHg) do not dominate parameters with smaller changes (e.g., temperature in °C). Every vital-sign measurement, *x*, was normalised using a zero-mean, unit-variance transformation, $x_{n} = (x - \mu )/\sigma$, where $x_{n}$ is the normalised value and $\mu$ and $\sigma$ are the mean and standard deviation of the vital sign, respectively, in the overall dataset (171 patients).

The Sammon mapping algorithm was then applied to the 770 normalised vectors contained in the 5 subgroups (G_1_, G_2_, G_3_, G_4_ and G_5_) from the 154 patients included in the “normal” subset of patients.

### Model of normality

We now consider the construction of a model of normality, based on all observations made on the last day on the ward (discharge day) of each patient from the “normal group”. This dataset contains the vital signs from the most physiologically stable period of the patient stay, because these data were acquired immediately prior to discharge from the ward, when the patient is at their most “normal” after recovering from surgery. This set of “normal” pre-discharge data contains 1,100 vital-sign vectors, **X** ∈ ℝ^5^, which were subsequently used for the construction of our model of normality.

A kernel density estimate [3] is a technique that allows the underlying 5-dimensional vital-sign pdf to be estimated from training data. A kernel density estimate was chosen because it is a non-parametric method, so no a priori assumptions are made about the form of the probability distribution.

The pdf of the set of *N* = 1,100 “normal” vectors, ${\mathbf{x}}_{{\mathbf{1}}} , \ldots ,{\mathbf{x}}_{N}$, was estimated using the following equation:

5 $$p({\bf x}|{\bf x}_{i} ,\sigma^{2} ) = \frac{1}{{N(2\pi )^{D/2} \sigma^{D} }}\sum\limits_{i = 1}^{N} {e^{{ - \frac{{\left\| {{\bf x} - {\bf x}_{i} } \right\|^{2} }}{{2\sigma^{2} }}}} } $$

which is a weighted sum of Gaussian kernels centred on the 1,100 vectors, ${\mathbf{x}}_{i}$, and where each kernel is isotropic with variance $\sigma^{2}$. The variance was determined using the nearest-neighbour method proposed by Bishop [2], in which the average of the squared Euclidean distance to the set of 10 nearest neighbours $\{ NNs\}$ is determined for each point ${\mathbf{x}}_{{\mathbf{1}}} , \ldots ,{\mathbf{x}}_{N}$ in ${\mathbf{X}}$,

6 $$\Updelta_{i} = \frac{1}{10}\sum\limits_{{j \in \{ NNs\} }} {\left\| {{\bf x}_{i} - {\bf x}_{j} } \right\|} $$

and $\sigma^{2}$ is estimated by calculating the average over all points:

7 $$\sigma = \frac{1}{N}\sum\limits_{i = 1}^{N} {\Updelta_{i} } $$

The likelihood for all data from the “normal” group of patients was then calculated using (5). The likelihood of all data from the “abnormal” group of patients, prior to the occurrence of an adverse event (either death or ICU admission) was also evaluated using the same model of normality.

In order to estimate the “abnormality” of a data point ${\mathbf{x}}$, the departure from normality is usually quantified using a novelty score defined as follows

8 $$z({\mathbf{x}}) = - \log p({\mathbf{x}}|\theta ) $$

where $z({\mathbf{x}})$ is the novelty score and $\theta = \{ {\mathbf{x}}_{i} ,\sigma \}$. “Normal” data, which have higher likelihoods $p({\mathbf{x}}|\theta )$, therefore generate low novelty scores $z({\mathbf{x}})$; conversely, “abnormal” data, which have lower likelihoods, generate high novelty scores $z({\mathbf{x}})$.

## Results

### Vital-sign distributions

**Fig. 2:**
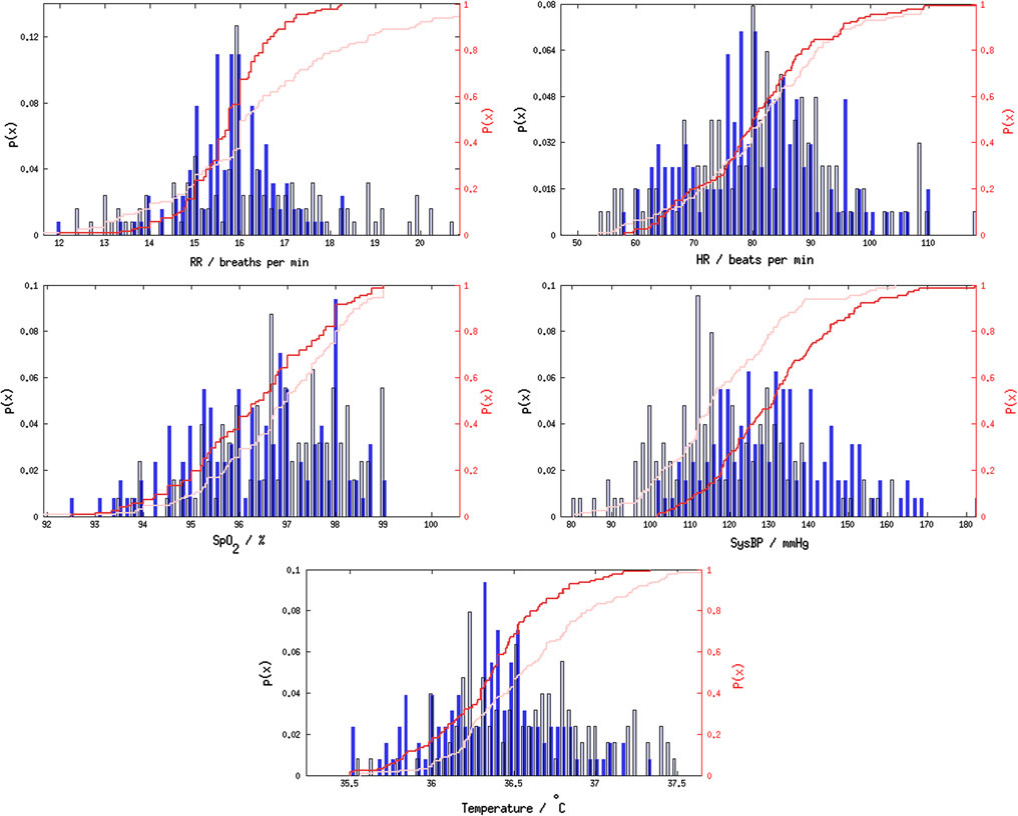
Histograms for respiratory rate, heart rate, blood oxygen saturation and temperature, computed from the average of vital-sign data acquired from patients at admission to the post-operative ward (*light blue*) and time near discharge (*dark blue*). Cumulative distribution functions $P(x)$ for each vital sign from patients at admission (*light red*) and time near discharge (*dark red*), are also represented (refer to the right vertical axis) (colour figure online)

Empirical pdfs (histograms) and cdfs for each physiological variable for each of the two subgroups G_1_ (average of observations at admission) and G_5_ (average of observations at discharge day) are shown in Fig. 2. Table 2 gives the corresponding means and standard deviations.

**Table 2:** Vital-sign means (SD) for admission and discharge days for “normal” patients

	RR (breaths per minute)	HR (beats per minute)	SpO_2_ (%)	SysBP (mmHg)	Temp. (°C)
Admission	16.7 (2.5)	80.6 (12.8)	97.0 (1.3)	115.7 (16.0)	36.6 (0.5)
Discharge	15.7 (1.0)	81.2 (11.7)	96.3 (1.5)	132.1 (16.6)	36.4 (0.4)

**Fig. 3:**
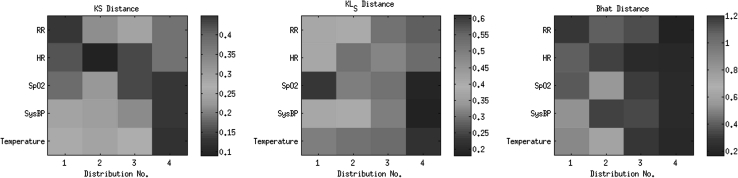
Representation of the distances between each of the 4 groups (G_1_, G_2_, G_3_ and G_4_ represented in the horizontal axis as *1*, *2*, *3* and *4*) and the G_5_ group. The Kolmogorov–Smirnov (*KS*), the symmetrical Kullback–Leibler (*KL*_S_) and the Bhattacharyya metrics for each distribution are shown in the form of matrices, in which the colour is associated with the values of the calculated distances (colour figure online)

Figure 3 shows the KS, KL and *Bhat* distance-maps between each of the distributions, for the 4 subgroups (G_1_, G_2_, G_3_, and G_4_) and the distribution for the G_5_ subgroup. In each distance-map, the subgroups involved (G_5_—G_*i*_ with *i* = {1, 2, 3, 4}) are represented on the *x*-axis, and physiological variables are represented on the *y*-axis. The colour code is associated with the values of the calculated distances (blue indicates small distances, and red indicates large distances).

It may be seen that, apart from the HR, the distributions represented for each of the other 4 vital signs vary from admission to discharge, as the patient recovers from surgery.

The results obtained by the three different metrics are very similar, in the sense that the patterns in the distances for each physiological variable are identical. The distances between the G_1_ and G_5_ distributions are greater than the distances between the G_3_ and G_5_ distributions.

### Data visualisation

**Fig. 4:**
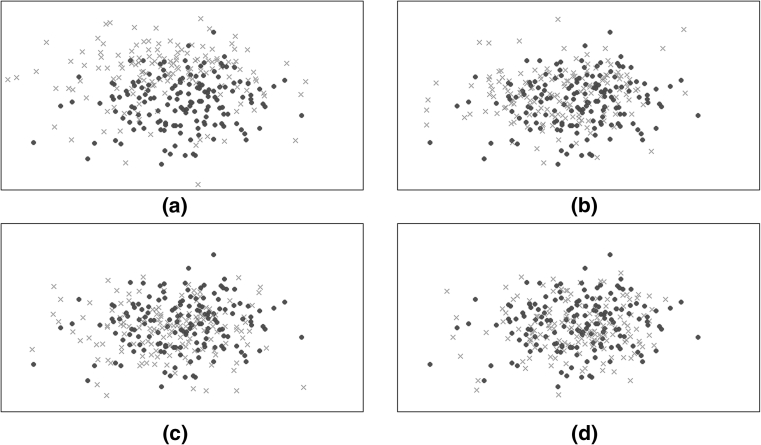
Sammon maps obtained for the groups: **a** G_1_ and G_5_, **b** G_2_ and G_5_, **c** G_3_ and G_5_, and **d** G_4_ and G_5_. Projected data from G_5_ are shown by *blue* $\bullet$; projected data from all other groups are shown by *red* ${\mathbf{x}}$ (colour figure online)

The resulting Sammon maps obtained are shown in Fig. 4. Represented in each map are the projected data points from G_1_, G_2_, G_3_, G_4_ (red crosses) superimposed on the projected data points from G_5_ (blue points).

### Model of normality

**Fig. 5:**
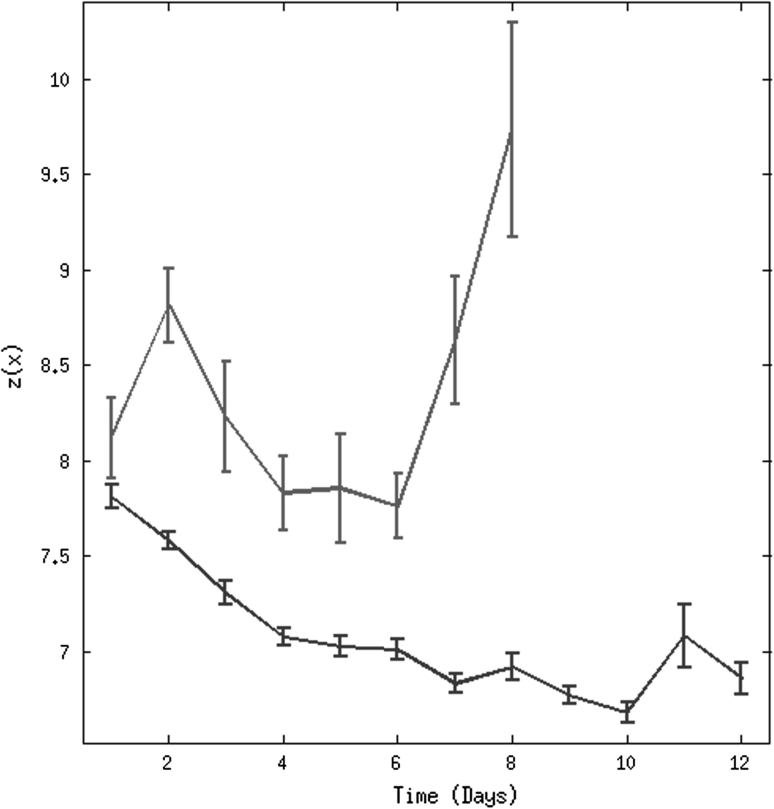
Representation of average (per day) of novelty scores $z({\mathbf{x}})$ against time for the “normal” group of patients shown in *dark blue* and the “abnormal” group of patients shown in *dark red*. *Error bars* denote one SE of the group mean (colour figure online)

The novelty scores are computed each day for each patient, by averaging the scores for each set of vital-sign observations that day. The group mean novelty scores $z({\mathbf{x}})$ for each day are shown in Fig. 5 for “normal” and “abnormal” patients. The median length of stay on the ward for the “normal” group of patients is 9 (see Table 1). For the “abnormal” group, we considered the length of stay on the ward prior to the event (either admission to the ICU or death). The median time to event for this group is 5 days. The novelty scores are displayed in Fig. 5 for the length of stay (or time to event in the case of the “abnormal” group) up to the 75th percentile (12 and 8 days, respectively) for each group of patients.

**Fig. 6:**
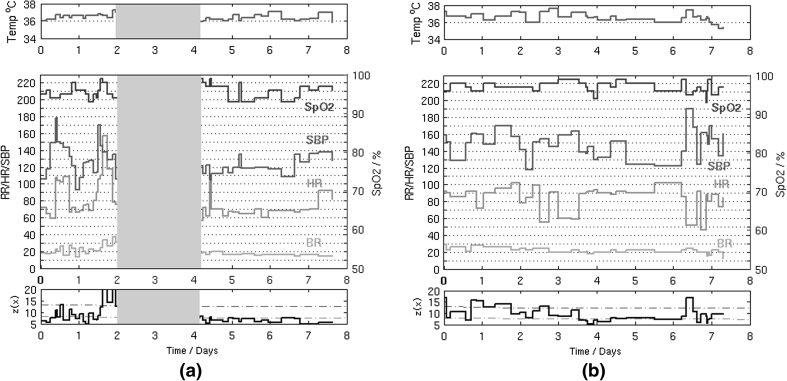
Two example patients are shown in (**a**) and (**b**). The *upper plots* show the observations of vital signs with time: temperature is shown in the upper plot in each column; RR, HR, SysBP and SpO_2_ are shown in the *middle plot* of each column (refer to the right vertical axis for SpO_2_). The *lower plot* in each column shows the novelty score $z({\mathbf{x}})$ determined using the model of normality: the decision threshold (*dashed red line*) was determined from the average of the density estimates for “normal” patients (*dashed green line*). The *grey box* in (**a**) indicates the patient’s admission to the ICU, during which period no vital-sign data are available (colour figure online)

Figure 6 shows the change in novelty score over time for two example patients from the “abnormal” group who deteriorate sufficiently after surgery to be re-admitted to ICU. A threshold $z({\mathbf{x}}) = k$ was determined using $k = \mu + 3s.d.$, where $\mu$ is the average of the density estimates $z({\mathbf{x}})$ for the 154 “normal” patients in the model of normality, and where $s.d.$ is one standard deviation of $z({\mathbf{x}})$ for these “normal” patients.

The first example (Fig. 6a) shows a patient who deteriorated 2 days after admission to the upper GI ward and was then admitted to the ICU. The patient was sent back to the upper GI ward after 2 days in the ICU. The patient stayed a further 4 days before being discharged. During the first 2 days after surgery, the patient exhibits physiological instability (which is more significant at the end of the second day) showing indications of tachycardia (HR reaching 150 beats per min) and tachypnea (RR reaching almost 40 breaths per min). It can be seen that $z({\mathbf{x}})$ increases in value at approximately 12 h before ICU admission, indicating physiological deterioration. After the stabilisation of the patient in the ICU, $z({\mathbf{x}})$ remains close to the “normal” trajectory.

The second example (Fig. 6b) shows a patient who had some periods of instability after being admitted to the upper GI ward, following surgery. After 7 days, the patient was re-admitted to the ICU, and then died 1 month later. In this case, large variations are observed in $z({\mathbf{x}})$ during the post-surgical period of abnormality, which are caused by episodes of elevated blood pressure (SysBP at around 190 mmHg) and bradycardia (HR decreasing to 45 beats per minute). These exceed the threshold defined by $z({\mathbf{x}}) = k$.

## Discussion

The histograms and cdfs shown in Fig. 2 indicate that the HR distributions are similar and approximately symmetrical. The distributions for SpO_2_ are one-sided because they are limited to the maximum value SpO_2_ = 100 %. For the distribution of SpO_2_ values at admission, a mode occurs at SpO_2_ = 97 %. Patients are likely to achieve 100 % oxygen saturation only if they are receiving additional oxygen through an oxygen mask. Therefore, the distributions shown in Fig. 2 exclude values of SpO_2_ >99 %. RR distributions are similar between admission and discharge. Tympanic temperature and SysBP distributions show that patients are, in general, mildly pyrexic (high temperature) and hypotensive (low systolic blood pressure) when admitted to the ward following surgery. They subsequently show decreasing temperature (back to “normal” values) and increasing blood pressure (back to “normal” values) by the last day of their stay on the ward.

From the distances between the distributions calculated with the three different metrics (Fig. 3), we can easily see the pattern of recovery with time: the distance between the G_1_ and G_5_ distributions is greater than, for example, the distance between the G_3_ and G_5_ distributions. If we consider the SysBP, for example, the KS, KL and *Bhat* distances between the G_1_ and G_5_ distributions are 0.29, 0.41 and 0.54, respectively, whereas the distances between the G_3_ and G_5_ distributions are 0.21, 0.31 and 0.34.

The Sammon maps represented in Fig. 4 show that the projected data from the five groups form clusters with some overlap between them, but that there are groups with visually separable distributions. The G_1_ cluster is the most diffuse (shown in red, in the upper-left plot in Fig. 4), while the projected data from G_3_, G_4_ and G_5_ are more concentrated, and similar to each other in their locus in the projection plane. This suggests that there are no large changes in the vital-sign distributions from halfway through a patient’s stay to the time of their discharge from the ward. That is, “normal” patients appear to have stabilised at around halfway through their stay on the ward. These results suggest that patients included in the “normal group” could have been considered for earlier discharge, or provided with a lower level of care from halfway through their stay.

From the trajectory of $z({\mathbf{x}})$ for the “normal” group of patients (Fig. 5a) we can see a significant decrease in $z({\mathbf{x}})$ in the first 4 days, after which $z({\mathbf{x}})$ is approximately constant for *t* ≥ 4 days. The first 4 days correspond to patient recovery immediately following surgery [24]. After day 4, the majority of patients included in the “normal” group appear to have fully physiologically recovered from surgery and are physiologically stable. It could be argued that these patients are sufficiently stable for early discharge to be considered, or for them to be provided with a lower level of care should they need to remain in hospital for reasons not related to physiological instability. Conversely, $z({\mathbf{x}})$ for the “abnormal” group of patients, suggests that the physiological trajectory for these patients is significantly different to that of “normal” patients with a sudden increase in novelty in the last 48 h, following the gradual decrease prior to this. These results suggest that patients’ criticality could be assessed by evaluating the distribution of their vital signs using the novelty score of Eq. (8) after their admission to the post-operative upper GI ward, following major surgery.

In summary, this study indicates that multivariate models of normality may be used to assess post-operative patients’ criticality. A multivariate model of the distribution of vital-sign data from “normal” patients was constructed using a kernel density estimate, and tested using “abnormal” data from patients who deteriorate sufficiently after surgery to be re-admitted to the ICU. Significant differences were found between the physiological trajectories for “normal” patients and those for “abnormal” patients.

## Footnotes

1 The Glasgow Coma Scale is a scale from 3 to 15, in which 15 indicates “alert” consciousness and 3 indicates complete unresponsiveness.
